# 39 years of directional wave recorded data and relative problems, climatological implications and use

**DOI:** 10.1038/sdata.2018.139

**Published:** 2018-07-17

**Authors:** Angela Pomaro, Luigi Cavaleri, Alvise Papa, Piero Lionello

**Affiliations:** 1 Institute of Marine Sciences, 30122 Venice, Italy; 2 Centro Previsioni e Segnalazioni Maree of the Municipality of Venice, 30100 Venice, Italy; 3 University of Salento, 73100 Lecce, Italy; 4 Euro-Mediterranean Centre on Climate Change, 73100 Lecce, Italy

**Keywords:** Physical oceanography, Physical oceanography

## Abstract

The dataset consists of 39 years of directional wave time series recorded since 1979 at the CNR-ISMAR “Acqua Alta” oceanographic research tower, located in the Northern Adriatic Sea. The extent of the time series allows us to describe the wave climate in the North Adriatic region and to identify trends and links with large scale climate patterns obtained from a single and permanent observational source. The northern part of the Adriatic Sea is characterized by two main wind and correspondingly wave regimes, strongly forced by the regional orography. The high sensitivity of this particular area to even small variations of large scale meteorological patterns allows to explore possible links of the local wave, hence wind, activity with large-scale north hemisphere circulation or weather regimes. Different wave gauges have been used since the start of the measurements, progressively upgraded and repositioned during maintenance operations. The recorded wave data have been thoroughly verified and corrected where necessary.

## Background & Summary

**Figure 1:**
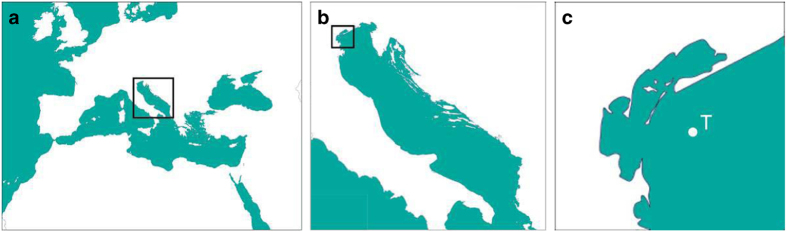
The area of interest. (**a**) The Adriatic Sea within the Mediterranean Sea, as part of the European region. (**b**) The Adriatic basin, about 750 km long and 200 km wide. (**c**) The Venice Lagoon and the oceanographic tower position T located about 15 km off the coastline (GPS coordinates 45° 18’ 51.288’’ N, 12° 30’ 29.694’’ E). Each small rectangle marks the area enlarged in the following panel.

The Acqua Alta oceanographic tower is located in the Northern Adriatic Sea, in 16 meters of depth, 15 km off the coast of the Venice lagoon (Fig. 1, GPS coordinates 45° 18’ 51.288’’ N, 12° 30’ 29.694’’ E). The tower is managed by the Institute of Marine Sciences of the National Research Council of Italy and is still in fully working order. A thorough upgrade of the structure has been recently completed, allowing the continuation of the described time series and further implementation of new wave measuring instruments. The importance of a proper measurements planning for long-term monitoring is taken into account.

The tower and its management were established following the disastrous flooding of Venice on November 4^th^, 1966. Unprecedented damages were reported all along the northern coast, where high waves had been superimposed on the almost two meter surge. A full hindcast of the storm and its flooding is provided in literature^1,2^. The effect of waves (set-up) in further rising the sea level at the coast has also been amply described^3^.

**Figure 2:**
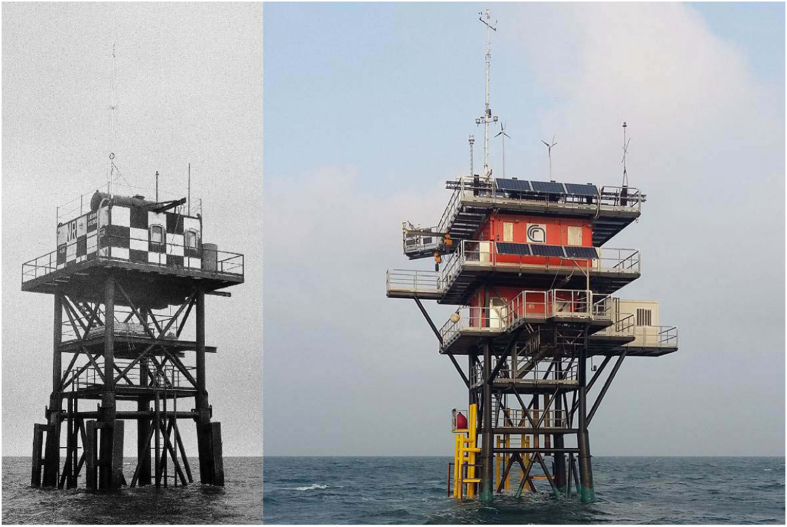
The Acqua Alta oceanographic tower. (**a**) In 1970, soon after its positioning, and (**b**) in 2017.

The tower was installed in March 1970 (Fig. 2) and dedicated special purpose wave measurement campaigns started in 1971. However, only in early 1979 regular automatic wave measurements were started. During the years, in response to the steadily growing use of the structure for meteorological, oceanographic, biological and chemical measurements, the tower has gone through various modifications and expansions, until the present configuration shown in Fig. 2.

Nowadays, the wave measurements continue on a regular basis, with the aim of better characterizing the wave climate in the Northern Adriatic Sea, with specific consideration for the Venice littoral, and of monitoring its long term wave changes.

Since the start of the measurements at the Acqua Alta research tower, the instrumental system has been progressively upgraded and repositioned during maintenance operations, and three different recording periods can be considered. In the first one (1979–1986) two pressure transducers were used. In the central period (1987–2003) the system was upgraded to three pressure transducers. In the third period (2004 to present) the pressure transducers have been replaced by echo sounders. Details are described in the Data Records section.

Issues related to lack of homogeneity in the recorded time series are discussed in the Methods session and methods leading to a corrected time-series are described. Granted the discontinuities and lack of the original raw data, the uniqueness of such a long wave dataset justifies the effort to make it available to the scientific community, together with the careful and documented analysis of the inhomogeneities and how they were removed, but providing the necessary details for proper dataset handling.

The dataset stands as one of the few multi-decadal observational wave time series beginning in the late 1970’s. At present, multi-decadal time series of wave data needed for climate studies are generally provided by long term model simulations (hindcasts) covering the area of interest. Examples, among many, at different scales are wind and wave hindcasts out of the ERA-Interim reanalysis of the European Centre for Medium-Range Weather Forecasts (ECMWF, Reading, U.K.). This has been used both at the global level and for regional re-analyses as for the Mediterranean Sea^4^. Valuable as they are, these estimates are necessarily affected by the approximations involved, the more so because of the problems encountered with modelling processes in small basins using coarse resolution wind fields^5^. Besides, following the availability since 1991 of altimeter data, and the consequent different quality of the analysis fields, the related discontinuity needs to be taken into account. On the contrary, multi-decadal observed time series have the evident advantage of representing the real evolution of the wave field, without the shortcomings associated with the limitation of models in reproducing the actual processes at work and their intrinsic variability. Obviously, observed wave time series are not exempt of problems. Apart from the accuracy of the measurements, they represent a local information, hence their use to describe the wave evolution at large scale is sometimes arguable. In general, this requires the support of additional studies assessing to which extent the local values are representative of large scale patterns. Moreover, a regular maintenance, accurate monitoring and metadata information are crucial issues when considering the reliability of a time series for climate applications. Unfortunately, similar datasets are rare, because in most of the times the aim of these measurements in the past did not envisage a long-term commitment. This makes the available dataset at the Acqua Alta oceanographic tower very useful and particularly interesting. Incidentally, together with the devoted keen measurement campaigns^6,7^ the long time series has allowed to delve further into the physics of the processes of interest. This is especially true if considering that waves, as an integrated product of the local wind climate, can provide related compact and meaningful information also on larger scale meteorological patterns.

## Methods

In this section we describe the measuring instruments and recording equipment that have been used during the measuring period. Issues related to lack of homogeneity in the recorded time series are discussed and the method leading to a consistent quality checked time series is explained.

The complete data record resulting from this work can be found at the link provided as Data Citation 1.

Operational wave measurements at the Acqua Alta oceanographic tower started in January 1979, continuing until the present time on an almost regular basis, with the aim of characterizing the wave climate in the Northern Adriatic Sea, with specific consideration for the Venice littoral.

Nowadays the extent of the dataset makes it interesting also from a climatological point of view, to monitor the regional long-term wave, hence wind, evolution. The first measurements started in early 1970’s with a pure scientific purpose. Regular recordings were begun in 1979, also with the aim of gathering information for a better design and management of the planned Venetian coastal defences, an action triggered by the cited disastrous storm of November 1966.

As already anticipated, different wave gauges have been used since the start of the measurements that we describe in the following subsections, referring to the three main periods.

### First period 1979–1986

In July 1978 a directional wave recording system was installed on the Acqua Alta oceanographic tower. Two small bore-hole, Bell&Howell, vented gauge pressure transducers were chosen as wave-measuring devices. Besides the limited cost of the instrument itself and the precision available, compared to other alternatives, a pressure transducer has several notable advantages: absence of sensitivity to surface breaking and air bubbles, sturdiness, reliability, limited power consumption and high resolution. In addition, its underwater position protects it from surface damage. The disadvantages of an underwater measuring system consist in the filtering of short, high-frequency waves and the sensitivity to fouling, hence the need for regular maintenance. Finally, the critical point is the necessity of relating the results to the surface displacement. This was done using transfer formulas derived from the linear theory^8^. If we assume a wave-length *L* on a bottom depth *d*, the related pressure attenuation factor Χ_*p*_ can be estimated as:

(1)χp=cosh[2π⋅(y+d)/L]cosh[2πd/L]

*y* being the depth of the considered point, positive upwards.

For the benefit of later discussion, for the local depth (16 m) and typical wave period (about 5-6 s), a 6 cm variation of instrument depth implies a 1% change of the recorded signal, which is the desired resolution for our wave measurements. It follows that $\chi_{p}$ is strongly dependent on the tide level, that must consequently be known with an error less than 6 cm. As later described, the depth of the transducers was derived from their signal. However, a further verification was done with the data of the on-board, highly accurate, fast response tide gauge^9^, capable to filter at better 1% all the waves with a period shorter than 10 s. See also for The related theory is amply documented in literature^10–13^.

A further problem arises from the dynamical effect of the orbital motion on the pressure transducer. This has been avoided by enclosing the transducer hole in a small soft water-filled plastic bag, thus reducing the dynamic pressure component to less than 1% of the signal^9,14^.

**Figure 3:**
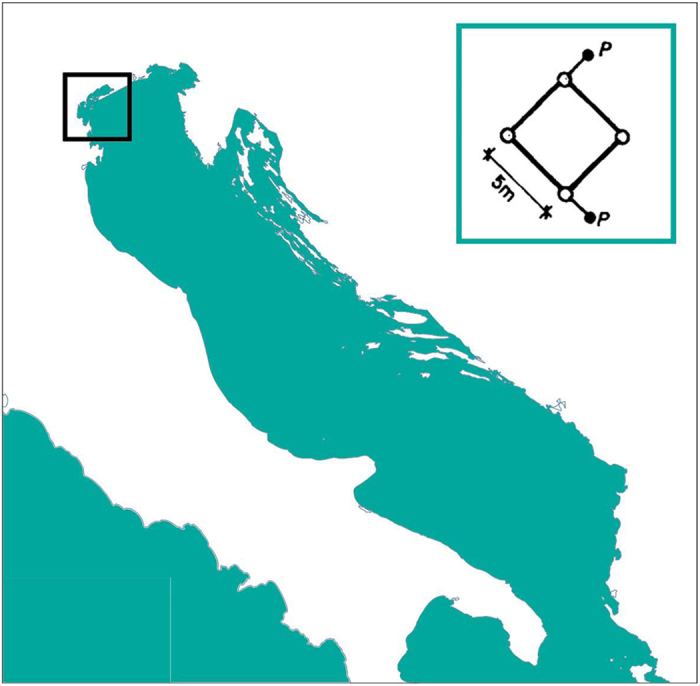
Position of the tower in the Adriatic Sea. The smaller figure shows its orientation and the location of the pressure transducers P.

The two transducers were placed at about 4 m depth, approximately 1 m below the deepest trough of the waves, 10 m aside, each 1.5 m away from the north and south legs of the tower (Fig. 3). The directional range from which relevant waves can approach the tower (see Fig. 1) is from 45° to 180° (geographical convention, clockwise with reference to the North). Using the phase differences, in the later spectral analysis this allowed to determine without ambiguity the mean direction for each frequency, hence the overall information. For shorter waves (typical mean period <3 s, hardly felt at 4 m depth) associated to local winds, hence possible also from land, the mean wave direction was assumed to equal that of the wind.

A pulley system has been used to place the transducers into their respective positions, allowing their easy recovery and resetting, without the need for diving during periodic inspection and cleaning operations.

The transducers were powered every three hours, at synoptic times. First, the system recorded for one minute. Evaluation of the overall excursion of the signal and comparison with a reference threshold led to the decision of either skipping the record or to proceed with a full 10 minutes data record at 2 Hz frequency. The time lag between the scan of the two transducers was 0.01 s. The data were then transferred to a computer-compatible incremental Kennedy tape recorder (model 1600, 7 tracks, 200 b.p.i., data rate 500 ch./s).

The range of the pressure transducers was 0÷25 p.s.i., with a full scale deflection of 5 VDC. Because of practical limits on local wave heights, this was reduced to 3.5, corresponding to a 12.4 m water column. Exceptional storm surges can raise the local sea-level up to +2 m that, together with the depth of the transducers and neglecting the wave attenuation with depth, allows almost 6 m of crest.

The resolution of the recording system corresponds to 1/1000, which equals 1.24 cm at the pressure transducer level.

In the data analysis phase, each record was first transformed into water height by appropriate calibration factors, then the average, representative of the mean depth of the transducer during the record, was subtracted assumed to be constant because of the limited tidal variation within the record duration (less than 3 cm).

If the estimated surface displacement was lower than 20 cm, the data were not considered and the record was skipped. If larger, the two series were Fourier analysed using the FFT technique. Routine analysis included evaluation of spectrum and cross-spectrum, then used to determine the related mean direction, actual attenuation of waves with depth and finally integrated parameters such as the significant wave height, wave period and mean direction.

### Second period 1987–2003

The use of only two transducers implied the lack of information on the directional spreading for each frequency band of the spectrum. Therefore in 1987 the system was upgraded with a third pressure transducer on the East leg, making directional spreading available for the analysis. The previous experience suggested to use absolute pressure transducers, the depth being established by their position and the on-board tidal signal. Also the tape unit originally used for storing the data was replaced with a solid state recorder.

The data acquisition system procedure did not change, but the recording duration was extended to 1024 s, with a sampling frequency of 1 Hz. A higher sampling rate was not considered because the corresponding wave frequency components would be completely attenuated at the pressure transducers installation depth, as it was also evident during the first period.

The resolution of the recording system corresponded to 1/256, which introduced a one unit white noise, showing up in the analysis as a white spectrum. Negligible with respect to the original pressure signal, if considered the noise would be strongly amplified in the high frequency range, leading to unrealistic shapes of the surface spectrum. This suggested a convenient cut-off frequency, after which, only for energy evaluation purposes, the spectra were fitted with a *f*^−5^ frequency tail^15,16^.

The procedure followed for the directional analysis is amply documented in literature, but it is qualitatively summarized below. Interested readers are referred to the quoted references for any specific formula.

The data analysis was based on the waves linear theory^8^, as successively modified and improved^17^. The directional analysis is based on the differences between the signals derived at the three sources. Starting from the three surface signals, applying standard cross-spectral analysis leads to 9 auto-, co- and quad-spectral density functions from which the first four Fourier coefficients of the directional distribution per frequency can be estimated as *a*_1_, *b*_1_, *a*_2_, *b*_2_. Then, according to the theory^17^, four physically sound definitions, free from model assumptions and computationally efficient, were assumed for the mean direction *θ*_*m*_, spread *σ*, skewness *γ* and curtosis *δ*, evaluated on the basis of the above coefficients. Hence, from each record the specific parameters *θ*, *σ*, *γ*, *δ* for each frequency, and the usual summarizing parameters of significant wave height *H*_*s*_, mean frequency (period) *f*_*m*_ (*T*_*m*_), and mean flow direction *θ*_*m*_ were derived.

This method cannot resolve bimodal directional distributions in the same frequency. However, it provides some checks to verify a possible bimodality in the input data, which do not affect the computational efficiency requirements of a routine analysis, with no significant loss of information. Alternative methods cannot be used because the original raw data are no longer available. After the data processing, only the integral parameters where archived. On a more physical basis, bimodality in the same frequency bin is not frequent in the Northern Adriatic Sea, as amply documented in the literature on the wave climate of the area. This is regulated by two main winds, namely bora and sirocco, acting practically at cross directions with large differences also from the perspective of wave generation. Bora is a cold, gusty and violent wind producing steep waves coming from North-East. Due to the short fetch of about 100 km, the wave period ranges between 5 and 7 s. Sirocco, from South-East, acts on a much longer fetch, up to 750 km. Less violent and more stable, it leads to longer, less steep waves, with typical periods between 7 and 10 s, often associated to storm-surge effects. Therefore, even when the two winds act together in the basin leading to cross sea conditions in the Northern Adriatic Sea, energy from multiple directions is not present in the same frequency range.

However, the long-term analysis revealed that an error had occurred in measuring the depth of the reference transducer. Hence, a dedicated procedure (see section Technical Validation) has been devised to obtain the best possible estimate to reconstruct the dataset for this period.

### Third period 2004 to present

The increasing need for maintenance led in 2004 to adopting external instruments, to be installed above the water surface. In order to check the new system, the data, obtained by echo sounders, have been checked with the ones retrieved by means of a parallel Acoustic Doppler Current Profiler (ADCP) system installed on the bottom, 20 m south-east of the tower. Because the echo sounder does not provide information on direction, this has been derived from the operational wave forecast model^18^ further verified with the ADCP data. Being the measurements now at the surface, a 4 Hz acquisition frequency is used. Of course this implies a different data availability for the high frequency part of the spectrum (i.e. very short waves) with respect to the two previous periods.

Varying air density, hence sound speed, is taken into account by means of a temperature transducer. The instrument is protected by direct solar radiation through an anti-radiance screen. The resolution of the signal is 1 cm.

From 2004 different recording periods have been used. Until 4^th^ September 2013 a three minute recording duration was used. From 4^th^ to 20^th^ September 2013 five minutes were used, after which the data recording duration has been extended to 15 minutes. In all these cases the data are available at 15 minute interval.

## Data Records

The data resulting from the analysis described in the previous section are available at the link provided as Data Citation 1. Starting in 1979, this dataset consists of a time series of the usual integral parameters as significant wave height *H*_*s*_, mean frequency and period, *f*_*m*_ and *T*_*m*_, peak frequency and period, *f*_*p*_ and *T*_*p*_ and the overall mean wave direction *θ*_*m*_.

The dataset analysis started with a careful reconstruction of the instrumentation type, installation details, data collection and archiving procedures and relative changes occurred along the years. It continued with several tests, aimed at proving, both from a physical and a statistical point of view, the validity of the reconstructed time-series. The partial correction required for the second period has been obtained via a reverse calculation process to derive the corresponding, no longer available, original pressure values. These were the starting point for the rescaling procedure, in so doing reducing any possible alteration effect on the data. The process has been validated through the careful analysis of the data statistical distribution, based both on waves linear theory and on the break-point investigation. The paper documents every passage of the reverse calculation process and of the statistical analysis performed on both the original and the “quality checked” dataset. This allows any researcher to understand the possible implications connected to the dataset handling with reference to the specific research purpose. Its relevance for long-term studies is also demonstrated in a paper which is based on the same dataset^19^. According to this paper, the correction introduced in the dataset does not emphasize the overall derived trends, because these are opposite to what implied by the data correction. Besides, the progressive wave climate evolution described by the “corrected” time-series is fully consistent with what derived from the analysis of only the first and third periods. This is consistent with the parallel analysis of the wind records. Here a distributed decrease of the maximum values is clearly visible, paralleled by an increase of the average storm intensity with a shift from the higher to the central part of the distribution. On the whole, the time series is available at 3 hour interval from 1979 to 2003. After this, beside the availability of recorded data at 15 minute interval, the associated 3-hour average values have been archived for each parameter. A higher recording frequency is desirable in an enclosed basin as the Adriatic Sea because the wave conditions can change rapidly, such as in case of bora storms, when, starting from a flat sea, the significant wave height can reach up to 3.0 m within one or two hours.

The present 39-year long (and still continuing), directional wave time-series is among the longest observational sources in the world. The implications of the described changes of instrumentation and maintenance operations have been explored via a critical analysis of the overall consistency.

A preliminary analysis revealed two breakpoints (see next session for details), corresponding to the equipment changes introduced in 1987 and 2004 and a significant inconsistency in the *H*_*s*_-*T*_*m*_ distribution for the second period, compared to the other ones. A keen analysis of the various possibilities has attributed this to an error in the depth originally reported for the reference transducer for the estimation of the actual *H*_*s*_. Being the support long term gone, a backwards procedure has been devised to estimate the correct depth with a consequent correction of the corresponding *H*_*s*_ and 1*D* spectral values.

The analysis has been based on monthly mean and percentile values of the parameters of interest, which are characteristics of the annual cycle and its variability, thus reducing noise effects associated to the daily variations.

The Buishand test^20^ has been used for the objective identification of breakpoints in the “raw” time series and for the validation of the procedure for their removal. This has helped the overall quality check of the dataset and made it suitable also for climatological analysis.

At this purpose and specifically for the identification of possible trends, the availability of a single long-term observational dataset is particularly relevant. A first study was carried out on this dataset, deriving the characteristic climatological trends associated to the *H*_*s*_ monthly percentile values. Also the correlation among the *H*_*s*_ time series and the region most relevant teleconnection patterns has been analysed according to the Pearson correlation coefficient^19^.

Note that a 4 Hz echo sounder system allows the detection, hence measurement, of also waves characterized by a mean period less than or equal to 3.0 s. The pressure transducers used in the two previous periods could not detect these data. Therefore, for the long-term analysis, in order to refer to homogeneous and comparable data, the cases with only very short period waves have been ignored also in the third period. The same logical approach has been followed for the record frequency, analysing only data at 3 hour interval^19^.

All the data handling has been performed using Fortran codes, as documented in the following Technical Validation section.

## Technical Validation

**Figure 4:**
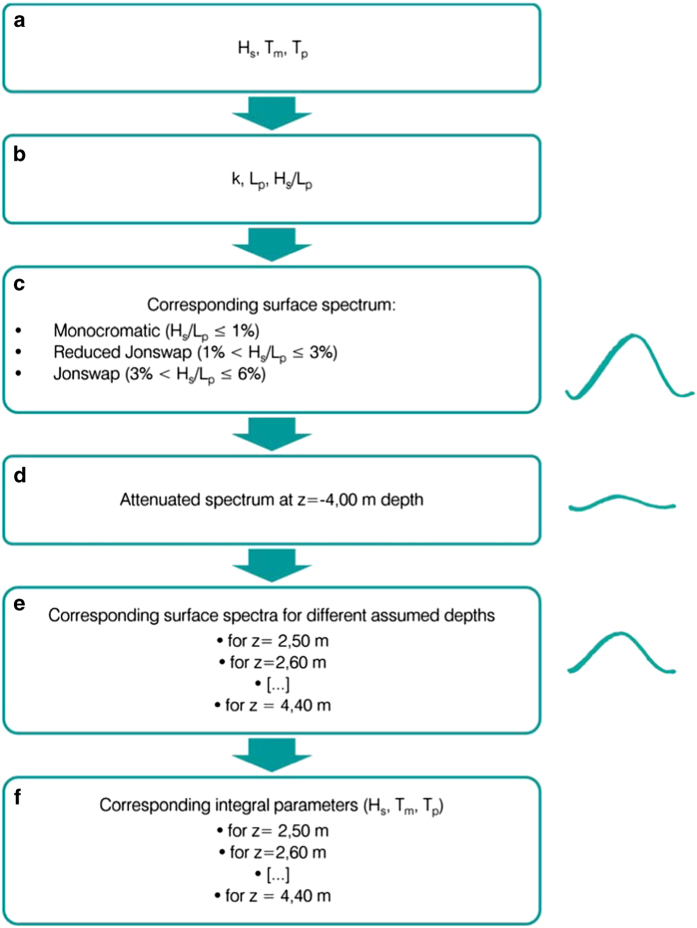
Block diagram of the re-scaling procedure applied to the second period. (**a**) Archived integral parameters, namely significant wave height *H*_*s*_, mean period *T*_*m*_ and peak period *T*_*p*_; (**b**) corresponding wave number *k*, wave length *L*_*p*_ and steepness $H_{s}/L_{p}$; (**c**) corresponding surface spectrum on the basis of three typical shapes derived from the wave steepness: monochromatic (for $H_{s}/L_{p}\leq 1\%$), reduced Jonswap (for $1\%<H_{s}/L_{p}\leq 3\%$) and Jonswap (for $3\%<H_{s}/L_{p}\leq 6\%$); (**d**) attenuated spectrum at 4,00 m depth; (**e**) corresponding surface spectra for different assumed depths (between z=−2,50 m and −4,40 m, at 10 cm interval); (**f**) estimate of the corresponding integral parameters *H*_*s*_, *T*_*m*_ and *T*_*p*_ for each possible depth.

As already anticipated, for the recognized discrepancy of the central period within the whole dataset, the corresponding data have been recomputed assuming a set of different values in the range 2.0 to 4.4 m as tentative depth of the transducers. The re-scaling procedure acts as a reverse calculation, that with some approximation reproduces the backward procedure used to derive the integral parameters from the pressure measure itself. The procedure is better explained with the block diagram provided in Fig. 4. The depth finally used for re-computing the corresponding correct time series, 3.0 m, has been identified as the one for which: 1) no break point is present in the overall timeseries; 2) the associated *H*_*s*_-*T*_*m*_ distribution is consistent with the ones of the first and third periods. In the following we describe the full procedure applied for the verification and partial correction of the original wave data.

**Figure 5:**
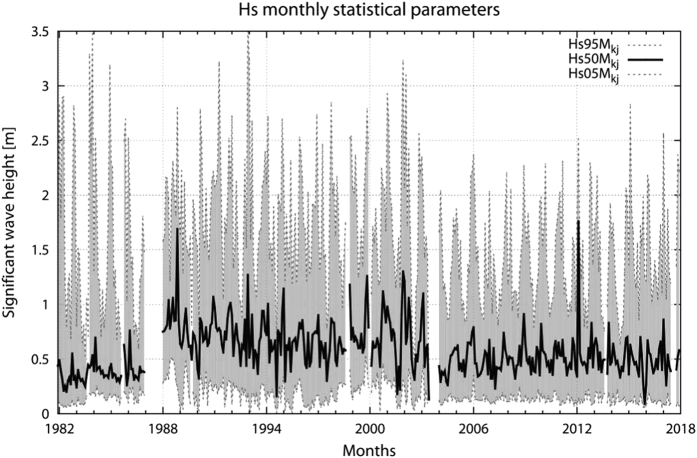
Statistical distribution of the 39-year long significant wave height original data set. The monthly values of the 5^th^, 50^th^ and 95^th^ *H*_*s*_ percentiles (Hs05Mkj, Hs50Mkj and Hs95Mkj) are given.

**Figure 6:**
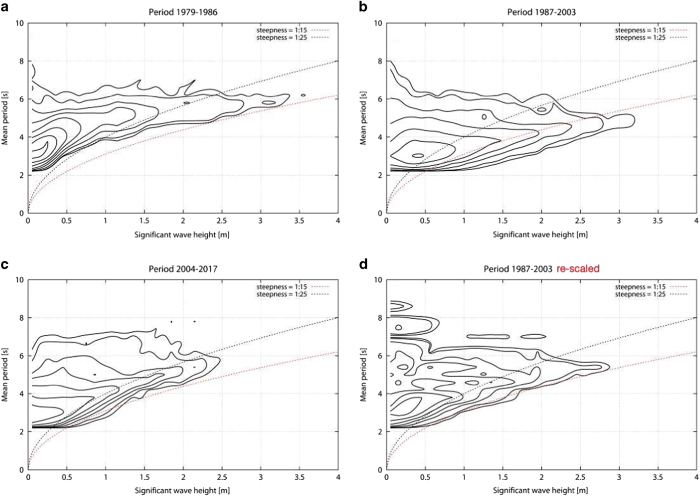
*Hs-Tm* statistical distribution for the measurement characteristic periods. Distribution for the periods (**a**) 1979-1986, (**b**) 1987-2003, (**c**) 2004-2015 and (**d**) for the period 1987–2003 after the rescaling procedure. Isolines at ratio 2, geometric progression. Dashed lines correspond to 1/15 and 1/25 steepness.

Figure 5 reports the *H*_*s*_ monthly percentiles (5^th^, 50^th^ and 95^th^) of the originally archived data along the whole 39-year long period. There is an evident inhomogeneity in the time series with higher wave heights concentrated in the central 1987-2003 period. The lack of homogeneity and the need to re-scale the data of the central period is also shown by plotting in Fig. 6 separately for each period the characteristic *H*_*s*_-*T*_*m*_ distribution^8^. The two dotted lines show the *H*_*s*_-*T*_*m*_ relationship for 1/15, 1/25 steepness respectively, defined as *H*_*s*_*/L*_*p*_, with *L*_*p*_ the dominant wavelength. These values represent the range above which wind sea data are extremely unlikely, or actually impossible. Smooth waves, not in generation conditions, group on the upper-left of these plots. Granted some differences due to the different measuring instruments (pressure transducers vs surface wave gauge, see the Methods section for the details), the distributions in panels *a* and *c*, respectively first and third period, comply with the cited upper limits of wave steepness, while the central, 1987-2003, period shows an evident presence of unrealistically steep waves. Therefore the monthly large values in the second period detected in Fig. 5 are not realistic and represent an error in the data-acquisition system.

The most likely explanation for the inhomogeneity was an error in the nominal depth assumed as a reference in the pressure transducer data processing. Clearly, the adoption of a depth reference value larger than the actual one would imply an overestimate of the signal attenuation at the transducer and a corresponding overestimate of the wave height at the surface. The error is strongly dependent on the signal characteristics, hence larger for short than for long waves, which results in a systematic overestimate of the mean wave steepness and *H*_*s*_, as indeed it is shown in the central part of the time series. The nominal depth of the east transducer, originally used for computing *H*_*s*_, had been assumed to be equal to 4.00 m with respect to the mean sea level, the actual depth being then derived by taking into account the locally measured tidal level. Any record or evidence of the actual positioning being since long term gone, a procedure was devised to identify the correct average depth.

A further evidence of the discontinuity has been given with the Buishand test^20^ applied to the originally archived *H*_*s*_ time series. This test marks the presence of an evident breakpoint at the beginning/end of the central period. Given a series of $x_{i}$ (i=1,n) values at regular time interval, the statistics are defined as:

$$S\left(k\right)=\sum_{i=1}^{k}\left(x_{i}-\overset{¯}{x}\right)/\sum_{i=1}^{n}{\left(x_{i}-\overset{¯}{x}\right)}^{2};k=1,...,n$$

where *S*(0)=0. For homogeneous time series, the values of S(k) oscillates around zero, because no systematic persistent deviation of the $x_{i}$ values with reference to the mean $\overset{¯}{x}$ is present for a long period. Therefore, in order to check whether a break is detectable at 99% or 95% significance level, we compute the Buishand test statistics by means of the *Q* statistics:

$$Q=\max_{0\leq k\leq n}S\left(k\right)$$

The relative *R* statistics (range statistics) is defined as:

$$R=\max_{0\leq k\leq n}S\left(k\right)-\min_{0\leq k\leq n}S\left(k\right)$$

hence function of the characteristic dataset length (*n*) and can be listed for different significance levels. In the present analysis, we refer to the $R/\sqrt{n}$ critical value correspondent to the 95% significance level, which equals 1.50.

This test has been applied also to the monthly percentile time-series.

A reference parameter, successively referred to as “breakpoint indicator” *IB*_*Bt*_, was computed to quantify the evidence of a discontinuity where relevant. *IB*_*Bt*_ is calculated as the sum of the number of detected breakpoints (at 95% confidence level) in the time series of the monthly 50^th^ percentiles weighted by a factor that varies with the distance in time when the instrumentation was changed. The factor equals 1.00 for breakpoints in the critical years, 0.67 at±1 year distance, 0.33 at±2, and 0 for any other eventually detected breakpoint. The results remain consistent when other percentiles are used. However, large percentiles tend to exhibits a large number of widespread spurious breakpoints, while small percentiles do not show strong evidence of discontinuities, consequently leading to a less robust identification of the actual breakpoints.

Starting from the original 1987–2003 data, derived assuming 4.00 m as the mean transducer depth, it is possible to recompute the *H*_*s*_ that would have occurred if the transducer depth had a different value (d≠4.00 m). The procedure consists of four main steps: a) estimating the original surface spectrum from each *H*_*s*_ and *T*_*p*_ pair, b) deriving from the reconstructed original surface values the corresponding signals at the assumed 4.00 m depth, c) deriving the surface spectra considering d≠4.00 m (see above) as the actual transducer depth, d) computing the corresponding *H*_*s*_ and *T*_*m*_.

In order to derive the spectrum starting from the integrated parameters (*H*_*s*_, *T*_*p*_ and *T*_*m*_) some assumptions have been made on its shape, depending on the wave steepness, i.e. if the sea could be considered under generation conditions (steep waves), or better represented as swell (smooth waves). The steepness *S* has been estimated as $S={H_{s}/L}_{p}$where *L*_*p*_ is the wavelength corresponding to the observed peak period. For values of S larger than 3% we have assumed a classical JONSWAP spectrum^21^ with the standard related parameters. For values of S between 1 and 3% we have used a “reduced JONSWAP”, where the JONSWAP spectrum has been scaled within each frequency range by a reduction factor *f*_*j*_:

$$f_{j}=0.25+0.75\cdot \frac{\left(steepness_{i}-0.01\right)}{\left(0.03-0.01\right)}$$

For values of *S* lower than 0.01 a very narrow spectrum was assumed, with energy concentrated within ±0.1*f*_*p*_ Hz of the peak frequency. The three ranges represent respectively active bora and sirocco storms, relaxed conditions when a storm is abating, and swell. Note that the procedure changes both the *H*_*s*_ and the *T*_*p*_ and *T*_*m*_ distributions, because of the frequency dependent attenuation with depth. As a result, the application of this procedure to each pair of *H*_*s*_*-T*_*m*_ data transforms them in two new values *H*_*s*_(*d*)−*T*_*m*_(*d*).

Essential requirement for the application of the described data check procedure to the original time-series is that both *H*_*s*_ and *T*_*m*_, hence *T*_*p*_, pairs are available. When the procedure encounters a limited number of failures with residual unrealistic values of steepness (*S*>6%, this corresponds to a minor percentage of the available data), the datum was rejected.

**Figure 7:**
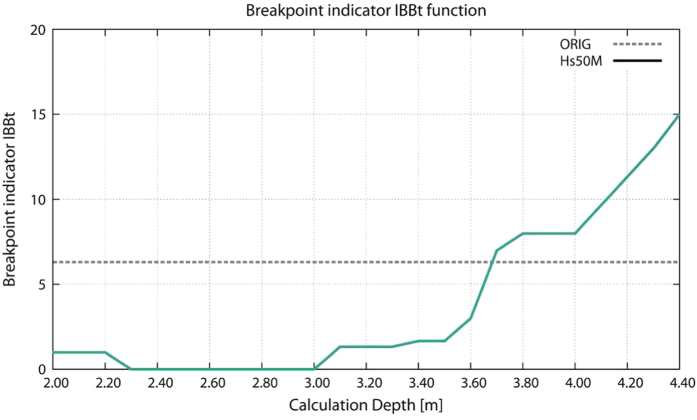
Breakpoint indicator *IB*_*Bt*_ (y-axis) as a function of the transducer assumed depth (x-axis values expressed in meter).

The procedure allows producing for the central 1987–2003 period a set of re-scaled time series, each of them corresponding to a different transducer depth. The correct transducer depth should cancel the break points at the beginning and end of the central period. Fig. 7 reports the values of the breakpoint indicator *IB*_*Bt*_ as a function of the assumed transducer depth. Together with the consequent *H*_*s*_-*T*_*m*_ distribution, this suggests the correct depth to be between 2.70 and 3.00 m.

**Figure 8:**
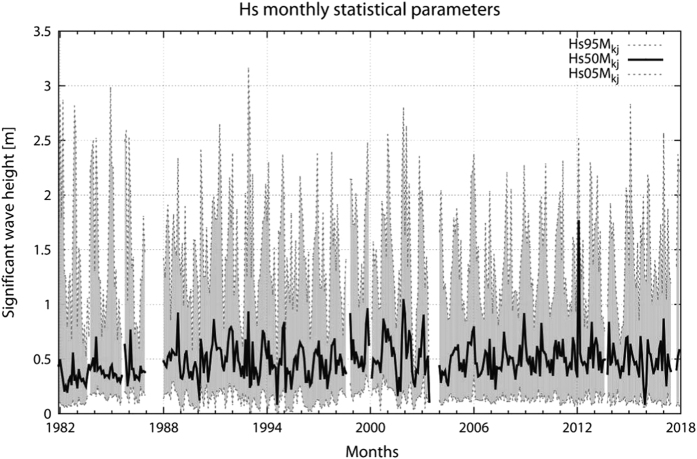
Statistical distribution of the 39-year long significant wave height quality checked data set. The monthly values of the 5^th^, 50^th^ and 95^th^ *H*_*s*_ percentiles (Hs05Mkj, Hs50Mkj and Hs95Mkj) are given.

On the basis of these considerations we have assumed 3.00 m (±0.05 m) as the correct depth of the transducer as this is the value with no breakpoints and the minimal difference with respect to the original nominal depth. The comparison between Fig. 8 and Fig. 5 shows the evident improvement, where no inhomogeneity is present.

The improvement is also evident when considering the statistical distribution of *H*_*s*_ in the three periods (Fig. 6), before and after the rescaling. The correction process procedure the agreement among the different periods and strongly reduces the fraction of high *H*_*s*_ in the central period.

## Additional information

**How to cite this article**: Pomaro A. *et al*. 39 years of directional wave recorded data and relative problems, climatological implications and use. *Sci. Data* 5:180139 doi: 10.1038/sdata.2018.139 (2018).

**Publisher’s note**: Springer Nature remains neutral with regard to jurisdictional claims in published maps and institutional affiliations.

## Supplementary Material
